# Physiological functions of glucose transporter-2: From cell physiology to links with diabetes mellitus

**DOI:** 10.1016/j.heliyon.2024.e25459

**Published:** 2024-01-29

**Authors:** Zhean Shen, Yingze Hou, Guo Zhao, Libi Tan, Jili Chen, Ziqi Dong, Chunxiao Ni, Longying Pei

**Affiliations:** a Xinjiang Institute of Technology, Aksu, China; b Sanquan College of Xinxiang Medical University, Xinxiang, China; c National Cancer Center/National Clinical Research Center for Cancer/Cancer Hospital, Chinese Academy of Medical Sciences and Peking Union Medical College, Beijing, China; d School of Laboratory Medicine and Biotechnology, Southern Medical University, China; e Department of Nutrition and Food Hygiene School of Public Health, Zhejiang University School of Medicine, Hangzhou, China; f School of Public Health, Peking University Health Science Center, Beijing 100021, China; g Hangzhou Lin ‘an District Center for Disease Control and Prevention, Hangzhou, China

**Keywords:** Glucose, Glucose transporter-2, Intestinal function, Diabetes, Signal transduction

## Abstract

Glucose is a sugar crucial for human health since it participates in many biochemical reactions. It produces adenosine 5′-triphosphate (ATP) and nucleosides through glucose metabolic and pentose phosphate pathways. These processes require many transporter proteins to assist in transferring glucose across cells, and the most notable ones are glucose transporter-2 (GLUT-2) and sodium/glucose cotransporter 1 (SGLT1). Glucose enters small intestinal epithelial cells from the intestinal lumen by crossing the brush boundary membrane via the SGLT1 cotransporter. It exits the cells by traversing the basolateral membrane through the activity of the GLUT-2 transporter, supplying energy throughout the body. Dysregulation of these glucose transporters is involved in the pathogenesis of several metabolic diseases, such as diabetes. Natural loss of GLUT-2 or its downregulation causes abnormal blood glucose concentrations in the body, such as fasting hypoglycemia and glucose tolerance. Therefore, understanding GLUT-2 physiology is necessary for exploring the mechanisms of diabetes and targeted treatment development. This article reviews how the apical GLUT-2 transporter maintains normal physiological functions of the human body and the adaptive changes this transporter produces under pathological conditions such as diabetes.

## 1 Introduction

**Fig. 1:**
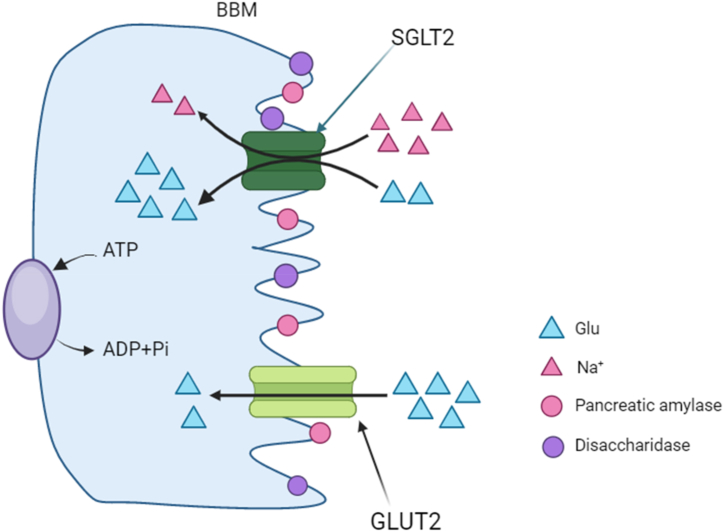
Schematic diagram of the action mechanisms of the glucose transporters.

Glucose is a common starting material for critical biochemical pathways, such as glucose metabolism and pentose phosphate pathway, playing a crucial role in energy conversion of the human body. From lactose in milk to high molecular weight carbohydrates, these substances will be broken down into simple sugars, such as glucose, fructose, and galactose, releasing energy. This process relies on a series of complex biochemical reactions and a series of catalytic enzymes, such as pancreatic amylase and disaccharidase, which are anchored initially on the surface of the brush border of the small intestine. Small intestinal epithelial cells at the tips of the small intestinal villi are responsible for monosaccharide transport. When transported, monosaccharides such as glucose must successfully cross the barrier of a dense layer of epithelial cells [1]. These cells contain tiny intercellular spaces produced by the tight junctions, creating a barrier that hinders glucose from entering the membrane pores [2]. Therefore, glucose entry relies on diffusion-promoting transporters (GLUTs) located in the luminal brush boundary membrane (BBM) and basolateral membrane (BLM) of small intestinal epithelial cells. It crosses the BBM via sodium/glucose cotransporter (SGLT1) into the cells and exits through the BLM using solute carrier family 2 (GLUT-2) transporter. In addition, this transporter moves fructose and glucose outside the enterocytes across the BLM under basal conditions [3]. The BLM in the jejunum of rats also contains a mechanism for rapid glucose regulation independent of the amount of GLUT-2 [4]. The SGLT1 transporter mainly depends on the Na^+^ concentration gradient and Na^+^-K^+^-ATPase-generated transmembrane potential (Fig. 1). When the glucose concentration in the small intestinal lumen is high, the GLUT-2 transporter is also embedded in the BBM, mediating glucose passage on both ends of the cells. Hence, these crucial molecules and enzymes form the spatiotemporal regulatory network in human intestinal cells.

Solute carrier family 2 (GLUT-2) has captured the attention of many scholars due to its tissue specificity and the complexity of gene expression regulation [5,6]. Its expression is in the intestine and liver of streptozotocin-induced laboratory mice with hypoinsulinemia and hyperglycemia, suggesting that both conditions enhance GLUT-2 expression. Conversely, its expression is downregulated in parenteral nutrition and diabetic pancreas experimental models [7,8], implying that factors besides blood sugar and insulin also affect GLUT-2 expression. When metabolic diseases (e.g., diabetes) occur in the body, the transporter undergoes compensatory function regulation, complicating GLUT-2 gene regulation. Emerging evidence reveals the potential role of GLUT-2 in diabetes pathogenesis, yet the underlying mechanisms remain unclear. This article reviews the role of GLUT-2 in intestinal physiology, related physiological regulation mechanisms, and pathophysiological changes in diabetes. It summarizes the recent advances in understanding GLUT-2 physiological functions and its role in diabetes mellitus. It mainly focuses on the older high-impact literature and novel impactful research that is generally well accepted to provide a new perspective on the pathogenesis and therapy of diabetes mellitus.

## 2 Basic overview of GLUT-2

### 2.1 Regulation of GLUT-2 gene expression

In human hepatocytes, GLUT-2 is a glucose-sensitive gene along with genes encoding type 1 pyruvate kinase and S14 fatty acid synthase [5]. In these cells, glucose-induced transcription is achieved by activating carbohydrate response element binding protein (ChREBP) transcription factor [9]. Xylulose 5-phosphate is an intermediate metabolite of the pentose phosphate pathway and an activator of the protease PP2A. This enzyme mediates ChREBP dephosphorylation and activation, promoting its transportation to the nucleus and transcription of glucose-sensitive genes [9]. Interestingly, the group that discovered this mechanism also found that the GLUT-2 promoter lacks the carbohydrate response element ChoRE and instead contains the sterol response element SRE, which also binds sterol regulatory element-binding protein 1 (SREBP-1C) [10,[17], [18], [19]]. This transcription factor controls the transcription of lipogenic genes that require simultaneous regulation of insulin and blood glucose concentrations, and in the pancreas, it regulates GLUT-2 transcription more strongly than ChREBP [5,18]. Therefore, GLUT-2 expression is regulated by blood glucose concentration and adipogenesis factors, although additional regulatory factors beyond the above may be involved, waiting for discovery. Evidence suggests some transcription factors, such as CCAAT/enhancer-binding protein alpha (C/EBP-alpha) and C/EBP-beta, that interact with the same sequence of the human GLUT-2 promoter and are involved in GLUT-2 expression, form dimers, enhancing GLUT-2 transcription in hepatocytes [9]. Similarly, the alpha subunit of the retinoic X receptor (RXR-alpha) binds to the gamma subunit of the peroxisome proliferator-activated receptor (PPARgamma), involved in the antidiabetic effect of thiazolidinediones, upregulating GLUT-2 expression in hepatocytes. In addition, hepatic transcription factor 1 (HNF1α) and forkhead box A2 (FOXA2) interact with the HNF sequence in the GLUT-2 promoter. In addition, hepatic transcription factor 1 (HNF1α) and forkhead box A2 (FOXA2) interact with the HNF sequence in the GLUT-2 promoter, increasing GLUT-2 expression [10,11]. The liver exhibits the highest expression of HNF1α, followed by the kidney and intestine, consistent with GLUT-2 distribution [12]. Interestingly, in hepatocytes, GLUT-2 expression is induced by glucose and fructose, not by the intermediate metabolites of glucose metabolism 2-deoxy-d-glucose or 3-*O*-methyl-d-glucose [13,14]. The reason for this regulation could be that GLUT-2 expression and pentose phosphate pathway have a common initiation mechanism.

Regulating GLUT-2 expression also seems crucial in other human organs, such as the brain and kidneys. Glucose transporters in capillaries and brain cells ensure adequate glucose provision to meet the energy requirements of cerebral neurons. In rodents, GLUT-2 expression occurs in several brain regions, such as the hippocampus, thalamic, hypothalamic, and brain stem nuclei (including the vagal motor nucleus and the nucleus of the tractus solitarius) [15]. The GLUT-2 protein in the brain regulates the glucose-dependent insulin and glucagon secretion [16], and in the BLM of proximal tubule cells allows glucose reabsorption from the glomerular filtrate in the kidneys. Knocking out GLUT-2 in mice and humans causes the excretion of filtered glucose in the urine, supporting its glucose reabsorption-stimulating effect.

In conclusion, the above investigations demonstrate that GLUT-2 and its regulatory network have the potential to impact various physiological functions in the human body.

### 2.2 Circadian regulation of GLUT-2

The expression of GLUT-2 mRNA in the small intestine is regulated by a circadian rhythm that changes light/dark conditions every 12 h. When GLUT-2 expression is observed in the mouse intestine, it peaks at the end of the light period and reaches a minimum at the end of the dark period [[19], [20], [21], [22]]. This expression dynamics may be due to vagus nerve regulation, inhibited absorption function of the intestine at night, and downregulated GLUT-2 expression by vagus nerve regulation [22]. Although the circadian rhythm of blood glucose-sensitive genes, such as GLUT-2, has been poorly studied in vivo, immunocytochemical staining of the islets of Goto-Kakizaki rats shows a gradual loss of β-cell GLUT-2 mRNA and elevated cytosolic soluble GLUT-2 protein levels [23]. However, 2 studies demonstrated that GLUT-2 is unlikely to be the principal glucose transporter in β-cells in humans and adult mice [[24], [25], [26]]. Indeed, GLUT-1 compensates for GLUT-2 loss as it occurs in beta TC3 cells and maintains a high capacity of glucose uptake to keep glucose metabolism in pancreatic β-cells [27]. Therefore, the role of β-cell GLUT-2 in regulating blood glucose levels and insulin secretion requires further validation. A detailed interaction of the expression of glucose transport vector with circadian and clock genes in the pig small intestine by Kinoshita Yuki et al. the study showed the levels of GLUT-2 mRNA exhibit significant diurnal variation that is always stronger in rats with a healthy metabolism. It also demonstrated significant diurnal changes in glucokinase mRNA, with higher levels of glucokinase mRNA in the pancreas of 6-week-old Landrace × Large White cross-breeds rats than in Wistar rats [28]. This effect causes glucose phosphorylation capacity to synchronize with food intake, enhancing glucose-stimulated insulin secretion and preventing postprandial hyperglycemia. Another investigation in a rat model assessed changes in gut tissue clock, gluconeogenesis, and glucose transport after duodenal-jejunal bypass (DJB) surgical intervention. It revealed that blood glucose levels were significantly lower in the rats who underwent DJB than in the sham-operated rats. The DJB procedure substantially induced GLUT-2 expression in the liver and elevated GLUT-2 and SGLT1 expression in the intestine. In addition, the expression of the cryptochrome circadian regulator 1 (Cry1) circadian transcription factor increased in the liver but decreased in the intestine of the DJB-operated rats. Similarly, the period circadian clock 2 (*Per2*) transcription factor was induced in the liver but repressed in the intestine of the DJB-operated rats [29,30]. These results suggest additional novel insights into the regulation of GLUT-2 gene expression.

### 2.3 Glucose-mediated regulation of GLUT-2 gene expression in the small intestine

The number of GLUT-2 transporters rapidly increases at small intestinal glucose concentrations of 30 mM and above, which depend on glucose-promoted short-term regulation of GLUT-2. When the glucose concentration is much higher than the Km of the SGLT1 transporter, the above process rapidly transports high concentrations of glucose into the cells, improving the absorption capacity of the small intestine for other monosaccharides, such as fructose [1,2,[30], [31], [32]]. When the insulin concentration in the lumen of the small intestine decreases, the abundance of GLUT-2 gradually decreases with time [33]. The translocation of GLUT-2 from the cytoplasm to the BBM mediates massive glucose absorption, while internalization of GLUT-2 from the BBM to the cytoplasm prevents postprandial hyperglycemia, conferring a dynamic balance [34]. Due to its uniquely low affinity for glucose (Km, 17 mmol/L), GLUT-2 plays a crucial role in various glucose-sensing cells, sampling a wide range of blood glucose concentrations. Studies have shown that in the short-term glucose-mediated regulation, GLUT-2 is mainly derived from vesicle exocytosis and embedded in small intestinal cells. However, the production and release mechanisms of the vesicles coupled with glucose concentrations have not been resolved. Some investigations have studied how GLUT-2 in vesicles inserts into the BBM, demonstrating that the protein kinase C (PKC) pathway may mediate the coupling of vesicle release and glucose concentration [35,36]. Mechanistically, the high concentration of glucose in the intestinal lumen of the small intestine first upregulates *SGLT1* expression to maintain the overall physiological needs of the organism. Furthermore, a high rate of SGLT1 transport depolarizes the BBM and further activates Ca^2+^ uptake [37,38]. Consequently, elevated intracellular Ca^2+^ concentration activates the PKC pathway to mediate myosin II phosphorylation [39,40]. The elevated Ca^2+^ driving myosin II contraction may rearrange the terminal reticulum, facilitating GLUT-2 insertion into the vesicle and the BBM [41]. In addition, resistin-like molecule beta (RELMb), a mediator of the mitogen-activated protein kinase (MAPK) pathway, promotes the recruitment of PKCβII to the plasma membrane and increases GLUT-2 levels in the BBM [38]. Several studies suggest that this process is related to glucose activating the paracrine pathway in enteroendocrine cells [41,42].

## 3 The links between GLUT-2 and diabetes pathogenesis

### 3.1 The role of GLUT-2 in the diabetes model

Mice subjected to a high-glucose diet for 30 days exhibit upregulated *GLUT-2* expression and accumulated GLUT-2 transporters in the BBM [43]. The levels of GLUT-2 can be quantified in a mouse model of diabetes using the hyperinsulinemic-euglycemic clamp. Mice are assigned to 2 groups and exposed to hyperinsulinemic-euglycemic clamp to assess GLUT-2 levels in the BBM. Normal mice injected insulin dispaly decreased GLUT-2 levels in the BBM. Remarkbly, the insulin-resistant mice remain the transporter levels. Moreover, these mice are resistant to insulin under the long-term high-sugar diet intervention. Mice treated with a high-glucose diet for 15 days under the hyperinsulinemic-euglycemic clamp and injected insulin display decreased GLUT-2 levels in the BBM. Remarkably, the transporter levels remain unaffected when the same technique is applied to insulin mice. Interestingly, the GLUT-2 transporter in the BBM can be observed within 30 min after feeding the mice with a high-glucose diet, and the levels of the transporter increase significantly. Before the high-sugar diet, the mice were exposed to insulin, and GLUT-2 levels in the mice were significantly lower than in the control mice. These experiments indicate that the pathologic enhancement of intestinal glucose absorption via GLUT-2 regulation plays a key role in postprandial hyperglycemia [44,45]. Furthermore, enhanced intestinal glucose absorption is present under diabetic conditions in mice, primarily due to the increased intestinal glucose transport capacity and permanent localization of GLUT-2 in the intestinal epithelial BBM [46]. Evidence also implies that insulin mediates GLUT-2 clearance from the BBM and attenuates glucose-dependent vesicular GLUT-2 transport [47,48].

### 3.2 GLP-1 and GLP-2 regulate blood glucose through GLUT-2

Glucagon-like peptide 1 (GLP-1) is a short peptide containing 30 amino acids and is mainly secreted by enteroendocrine L cells to regulate insulin secretion in the body [49]. An intravenous injection of GLP-1 mimics postprandial hormone concentrations, promoting insulin secretion and significantly inhibiting glucagon production in a dose-effect-dependent manner [50,51]. The abundance of the GLUT-2 transporter in the BBM can be reduced by insulin-induced GLP-1 secretion. The promoting effect of GLP-1 on insulin and the inhibitory effect on glucagon is glucose dependent. Glucagon-like peptide 2 (GLP-2) is a short peptide produced by dehydration condensation of 33 amino acids and is secreted by enteroendocrine L cells. Although it does not affect insulin production, it has a beneficial nutritional effect on the intestine and shares high structural similarity with GLP-1 [52]. This peptide promotes intestinal mucosal growth, inhibits gastric acid secretion, weakens the peristalsis of the intestine, and enhances GLUT-2 expression [[53], [54], [55]]. Meier et al. [56] have shown that GLP-2 promotes glucagon production in the body after intravenous injection. It also upregulates GLUT-2 expression and enhances the abundance of GLUT-2 transporter in the BBM [56]. The underlying mechanism may be related to GLP-2-dependent activation [57], where low insulin and high glucagon formed in type 1 diabetes promote GLP-2 secretion in the intestine, increasing GLUT-2 abundance in the BBM. The GLUT-2 transporter performs various other physiological functions, such as stimulating intestinal mucosal growth and resistance to intestinal barrier damage following an inflammatory response [[58], [59], [60]]. In a type 2 diabetes model, hyperglycemia, hyperinsulinemia, and proglucagon confer an opposite effect due to decreased GLP-2 expression. Consequently, a weakened intestinal barrier and worsened imbalance of intestinal flora cause sepsis, shock, and other grave medical conditions [61].

### 3.3 The possible mechanism of GLUT-2 affecting glucose absorption and glucose homeostasis

The GLUT-2 isoform is expressed in the liver, intestine, kidneys, pancreatic islet β cells, and in the cells of the central nervous system, such as neurons, astrocytes, and tanycytes [62]. Although it has a broad expression, GLUT-2 performs distinct physiological functions in different tissues. In the intestine, for instance, it is chiefly responsible for allowing glucose movement from the lumen toward the bloodstream [63]. In the liver, it is a critical factor for detecting glucose signals in the hepatic portal vein [64].

In the intestine, GLUT-2-mediated glucose diffusion is the main pathway of postprandial glucose absorption [63]. Moreover, because the intestine is the main source of blood glucose, inhibiting intestinal sugar digestion has become an attractive method to control postprandial hyperglycemia. Berberine is a customary component in Chinese medicine, characterized by diverse pharmacological effects, including anti-diabetes [65]. The GLUT-2 inhibitor phloretin, not the SGLT1 inhibitor phloridzin, blocks the effects of berberine on decreasing cellular glucose uptake under high glucose levels, indicating that GLUT-2 is the central target transporter mediating berberine-induced reduction of intestinal glucose absorption. Moreover, the BBM also reduces intestinal glucose absorption by inhibiting the IGF-1R-PLC-β2-GLUT-2 signaling pathway [46]. Hence, these 2 ways of lowering intestinal glucose absorption may represent novel strategies for the clinical treatment of hyperglycemic diseases, such as diabetes.

Furthermore, alongside glucose uptake, GLUT-2 expression considerably correlates with intestinal gluconeogenesis. This process was discovered in the 1990s and describes a close association with the stability of fasting glucose in human bodies [66]. It can also occur in overweight patients and those with insulin resistance following gastric bypass surgery [67]. When blood sugar is produced in the gut, it enters the bloodstream via 2 distinct routes: 1) a glucose-induced GLUT-2 translocation to the apical membrane to account for glucose absorption in response to a glucose load and 2) a GLUT-2 independent glucose export mechanism that depends on the previously phosphorylated glucose by hexokinases and dephosphorylation by glucose 6-phosphatase [66]. Intestinal gluconeogenesis is also linked with an individual's appetite, mood, and weight loss, as revealed by more recent studies [67].

In the hepatic portal vein, GLUT-2 plays an important role in glucose sensing. After a meal, glucose is absorbed in the small intestine and collected into the portal vein [68]. Glucose receptors sense the increased glucose concentration and send signals via the vagus nerve to target tissues throughout the body, particularly the heart and brown adipose tissue, and accelerate glucose uptake and use by the tissues and lower blood glucose concentrations [69]. The glucose sensing mechanism in the hepatic portal vein relies on the GLUT-2 transporter and was established by employing GLUT-2 knockout mice and conducting experiments using femoral and portal vein perfusion of glucose. Control mice exhibited hypoglycemia after portal vein infusion. By contrast, the GLUT-2 knockout mice showed transient hyperglycemia with glucose infusion through the portal and femoral veins, followed by a return to fasting glucose, similar to the performance of control mice with femoral vein infusion. These data indicate that a glucose sensor in the hepatic portal vein necessitates GLUT-2, whereas growth inhibitors function on cells that express GLUT-2 [70]. Furthermore, GLUT-2 is associated with glucagon release suppression, as revealed by a study on GLUT-2 knockout mice, where significant hyperglycemia was observed. In RIPGLUT1 [71]; GLUT-2^−/−^mice, the stimulation of glucagon secretion by hypoglycemia or its suppression by hyperglycemia was lost [72], although GLUT-2 was not expressed in pancreatic α cells. This observation implies that glucagon secretion regulation involves GLUT-2 expression in cells other than pancreatic α cells. Moreover, GLUT-2-deficient mice exhibited glucagon hyperglycemia that was subsequently normalized through nerve blockade, demonstrating the involvement of GLUT-2 in regulatory linkages associated with nerve signaling.

Mutations in the human GLUT-2 gene sequence are associated with glycogen storage defects in the kidneys and liver. A rare genetic GLUT-2 deficiency provokes a glycogen storage disorder called Fanconi-Bickel syndrome (FBS), which encompasses characteristic clinical signs such as fasting hypoglycemia and glucose and galactose intolerance [73,74]. However, GLUT-2 knockout mice are metabolically inconspicuous, suggesting that GLUT-2 abundance is not rate limited in murine metabolism [75]. Lactose intolerance is frequent in patients with FBS and results from impaired absorption of carbohydrates in the small intestine, causing diarrhea upon digesting carbohydrate-rich foods [76]. Sequencing the *GLUT-2* gene in samples from patients with lactose intolerance uncovered that single-nucleotide variants of *GLUT-2* were homozygous for inactive or truncated GLUT-2 transporters in 74 % of patients [[77], [78], [79]]. When *GLUT-2* homozygotic or compound heterozygotic for mutations cause FBS, glycogen accumulates in renal tubular cells, preventing them from reabsorbing various filtered solutes due to the impaired GLUT-2-mediated glucose efflux. These results suggest that GLUT-2 deficiency in FBS provokes high glucose accumulation in renal tubular cells [80]. A recent hypothesis proposes that GLUT-2 affects diabetes mechanisms by influencing glucose absorption. Specifically, GLUT-2 is elevated in the kidneys of patients with diabetes, enhancing glucose reabsorption and worsening hyperglycemia. When this condition was generated in mice with diabetes and obesity, and GLUT-2 was knocked out, the loss of GLUT-2 reversed hyperglycemia and normalized body weight [81]. Furthermore, this improvement in glycemic control was abrogated when GLUT-2 was knocked out in the kidneys and liver, suggesting that the improvement is attributable to GLUT-2 deficiency in the kidneys. In conclusion, renal GLUT-2 regulates systemic glucose homeostasis through glucose reabsorption, and deleting GLUT-2 is a potential therapy for diabetes and obesity [82]. Moreover, GLUT-2 plays a crucial role in human blood glucose regulation and may represent the central component for blood glucose regulation in the liver and kidneys.

As previously shown, mutations in the GLUT-2 gene result in excessive glycogen storage, mainly in the liver and kidneys. Previous case reports of this condition have described liver biopsies with glycogen stores and variable steatosis/fibrosis. Unlike other types of glycogen storage diseases, hepatocellular adenoma and carcinoma have not been described in this syndrome. A study in mice showed that blocking GLUT-2 translation in a specific neuronal population increases sugar-seeking behavior, highlighting the importance of GLUT-2 expression in the brain. This result also indicates that a decrease in GLUT-2 levels in the brain due to genetic polymorphisms or diseases affects health by modifying behavior. When the *slc2a2* gene, a functional ortholog of the human GLUT-2 gene, was knocked down in zebrafish to decipher the role of GLUT-2 in brain development, the *slc2a2* abrogation led to defective brain organogenesis, reduced glucose uptake, and increased programmed cell death in the brain. In addition, *slc2a2* deficiency affected the development of neural progenitor cells expressing the proneural genes atonal bHLH transcription factor 1b (*atoh1b*) and pancreas associated transcription factor 1a (*ptf1a*) but not those expressing neuronal differentiation 1 (*neurod1*), coinciding with the observed localization of *slc2a2* expression in the zebrafish hindbrain. These results indicate that GLUT-2 has an essential role during brain development by facilitating the uptake and availability of glucose and supporting its involvement in brain glucose sensing [83]. However, experiments in mice that investigated whether systemic reduction of a GLUT-2 allele has an effect on cognition revealed that the mice showing a systemic depletion of the GLUT-2 allele when fed a chow diet showed neurological functions and cognition of wild-type mice. This finding suggests that small changes in GLUT-2 levels occurring on a population level are unlikely to affect behavior and basic cognition [[84], [85], [86], [87], [88]].

## 4 Discussion and perspective

Continuous advance in global economy and rise in industrialization has considerably improved the socioeconomic conditions of the human population, yet at the same has increased binge eating habits and obesity. Obesity considerably increases the probability of individuals developing diabetes since obesity is a high-risk cause of the disease. Therefore, the demand for targeted small bowel interventions is becoming increasingly evident, and developing the related drugs is based on understanding the expression of human glucose transporters in small bowel function and their biological role in diabetes. In this study, we reviewed the normal physiological function of the glucose transporter GLUT-2 and its role in diabetes mellitus, with hope to identify gaps and find a new perspective for future research.

Many researchers have studied the role of transporters in monosaccharide transport and intestinal barrier integrity and obtained abundant experimental data. However, the specific biological mechanisms of these transporters remain unclear, demanding more in-depth studies to explain the function of monosaccharide transporters and refining experimental instruments. Moreover, the GLUT-2 transporter is not the only monosaccharide transporter in the intestine, further complicating the maintenance of glucose homeostasis and correlation with diabetes. For example, GLUT5 and SGLT1 are other known transporters in the small intestine, including several glucose transporters with unclear biological functions. Because the contribution of these transporters is unknown, the experimental data obtained from the existing relevant studies may be biased. Another drawback of these studies is that they do not consider the differences between short- and long-term post-translational regulation of the GLUT-2 transporter, use rodents as experimental models, and not take into account heterogeneity between humans and rodents.

## 5 Conclusion

GLUT-2 is a glucose transporter in the human body, playing a crucial role in maintaining the metabolic and energy homeostasis of the body, especially when it is in a hyperglycemic state. Our review highlighted the roles of the GLUT-2 transporter in maintaining glucose homeostasis in the human body and how dysregulations in this transporter cause metabolic diseases, such as diabetes. Some researchers believe that blocking the GLUT-2 transporter under hyperglycemia could be a potential target for achieving weight loss in patients with diabetes and obesity. However, the specific biological functions of GLUT-2 in diabetes remain unclear, and whether the transporter performs other physiological functions in the gut is still unexplored. Thus, the designed targeted drugs may cause metabolic disorders and damage the natural barrier function of the small intestine, causing an imbalance of normal intestinal flora [89,90]. Nonetheless, altering GLUT-2 activity is still a constructive drug design idea for treating diabetes. With deepening research on GLUT-2 and the precision of experimental instruments, our knowledge of GLUT-2 is becoming increasingly clear, opening the door to developing drug targets for diabetes.

## Funding

This study was supported by the Central Guiding Local Science and Technology Development Special Fund Project (No. ZYYD2022C03) and the College Student Key Innovation Planning Project of Henan Province (No. 202313505001).

## Ethics approval and consent to participate

Not applicable.

## Consent for publication

Not applicable.

## Data availability statement

Has data associated with your study been deposited into a publicly available repository?

Authors’ response: No.

Has data associated with your study been deposited into a publicly available repository?

Authors’ response: No data was used for the research described in the article.

## CRediT authorship contribution statement

**Zhean Shen:** Writing – review & editing, Writing – original draft, Supervision, Investigation, Formal analysis, Data curation, Conceptualization. **Yingze Hou:** Writing – review & editing, Writing – original draft, Software, Methodology, Funding acquisition, Formal analysis, Data curation. **Guo Zhao:** Writing – review & editing, Writing – original draft, Software, Project administration, Methodology, Data curation. **Libi Tan:** Writing – review & editing, Writing – original draft, Software, Methodology. **Jili Chen:** Writing – review & editing, Resources. **Ziqi Dong:** Writing – review & editing, Resources. **Chunxiao Ni:** Writing – review & editing, Resources. **Longying Pei:** Writing – review & editing, Supervision, Resources, Funding acquisition, Data curation.

## Declaration of competing interest

The authors declare the following financial interests/personal relationships which may be considered as potential competing interests:Peilong Ying reports was provided by Xinjiang Institute of Technology. If there are other authors, they declare that they have no known competing financial interests or personal relationships that could have appeared to influence the work reported in this paper.
